# Development and Validation of Stability-Indicating Method for Estimation of Chlorthalidone in Bulk and Tablets with the Use of Experimental Design in Forced Degradation Experiments

**DOI:** 10.1155/2016/4286482

**Published:** 2016-03-31

**Authors:** Sandeep Sonawane, Sneha Jadhav, Priya Rahade, Santosh Chhajed, Sanjay Kshirsagar

**Affiliations:** MET's Institute of Pharmacy, MET League of Colleges, Bhujbal Knowledge City, Adgaon, Nashik, Maharashtra State 422003, India

## Abstract

Chlorthalidone was subjected to various forced degradation conditions. Substantial degradation of chlorthalidone was obtained in acid, alkali, and oxidative conditions. Further full factorial experimental design was applied for acid and alkali forced degradation conditions, in which strength of acid/alkali, temperature, and time of heating were considered as independent variables (factors) and % degradation was considered as dependent variable (response). Factors responsible for acid and alkali degradation were statistically evaluated using Yates analysis and Pareto chart. Furthermore, using surface response curve, optimized 10% degradation was obtained. All chromatographic separation was carried out on Phenomenex HyperClone C 18 column (250 × 4.6 mm, 5 *μ*), using mobile phase comprising methanol : acetonitrile : phosphate buffer (20 mM) (pH 3.0 adjusted with* o*-phosphoric acid): 30 : 10 : 60% v/v. The flow rate was kept constant at 1 mL/min and eluent was detected at 241 nm. In calibration curve experiments, linearity was found to be in the range of 2–12 *μ*g/mL. Validation experiments proved good accuracy and precision of the method. Also there was no interference of excipients and degradation products at the retention time of chlorthalidone, indicating specificity of the method.

## 1. Introduction

A Stability-indicating assay method can be defined as “Validated quantitative analytical method that can detect the change with time in the chemical, physical or microbiological properties of the drug substance and drug products are specific so that the content of active ingredients and degradation products can be accurately measured without interference” [1].

Generally forced degradation/stress testing is used to generate the samples for stability-indicating assay methods. Forced degradation/stress testing is defined as “the stability testing of drug substance and drug product under conditions exceeding those used for accelerated stability testing” [2]. Degradation can be achieved by exposing the drug, for extended period of time, to extremes of pH (HCl or NaOH solutions of different strengths), at elevated temperature, to hydrogen peroxide at room temperature, to UV light, and to dry heat (in an oven) to achieve degradation to an extent of 5–20%. Generally, trial and error experimentation is used during these experiments. This trial and error approach is generally cost, labor, and time intensive and should be substituted with some systematic approach. From exhaustive literature, it was observed that experimental design in forced degradation experiments can be used to save cost and labor by avoiding trial and error experimentation [3, 4].

General steps involved in experimental design strategy are the selection of variables, selection of response, selection of design, preparation of experimental domain, preparation of experimental matrix, generation of polynomial equation, screening the important/most affecting variables using either one-way ANOVA or Pareto chart or using normal/half normal plots, and finally selection of optimum region by surface response.

The aim of the present work was to implement experimental design strategy in forced degradation experimentation and to arrive at optimum degradation conditions. For the same, chlorthalidone was selected as a model drug.

Full factorial experimental design was used during acid and alkali degradation experiments. After determining the polynomial equations, significant factors were determined using ANOVA and Pareto chart and finally the optimum regions for acid and alkali degradation conditions were determined using surface response curve.

**Figure 1:**
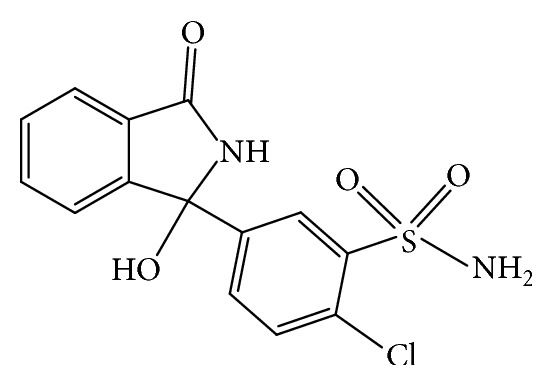
Chemical structure of chlorthalidone.

Chemically, chlorthalidone is 2-chloro-5-(2,3-dihydro-1-hydroxy-3-oxo-1H-isoindol-1-yl) benzenesulfonamide (Figure 1) [5]. It is an antihypertensive and diuretic drug used alone or in combination with other drugs to treat hypertension and various renal disorders. Literature survey revealed that few stability-indicating methods are reported for the estimation of chlorthalidone in combination with other drugs [6–8].

## 2. Materials and Methods

### 2.1. Chemicals and Reagents

Pharmaceutical grade chlorthalidone was supplied as a gift sample from Trichem Laboratories Ltd., Mumbai, India. Methanol and acetonitrile used in analysis were of HPLC grade and all other chemicals and reagents were of analytical grade and were purchased from SD Fine Chemicals, Mumbai, India. Double distilled water used was freshly prepared by Double Distillation Assembly (Borosil, Mumbai, India) and further used in analysis after filtering through 0.45 *μ* membrane filter papers purchased from Millipore (India) Pvt. Ltd., Bengaluru, India. Chlorthalidone tablets (label claim 12.5 mg/tablet) were purchased from local market.

### 2.2. Apparatus and Equipment

HPLC instrumentation consisting of pump PU-2080 plus (JASCO, Tokyo, Japan), with Rheodyne manual loop injector 7725*i* (injection loop capacity 20 *μ*L) was used. Detection was carried out using UV-2075 detector (JASCO, Tokyo, Japan). Data acquisition was done by Borwin chromatography software version 1.5 (JASCO, Tokyo, Japan). All calculations were performed using Microsoft Excel 2010 (Microsoft Corporation).

### 2.3. Chromatographic Conditions

All chromatographic separations were carried out on Phenomenex HyperClone C 18 column (250 × 4.6 mm, 5 *μ*), using mobile phase comprising methanol : acetonitrile : phosphate buffer (20 mM) (pH 3.0 adjusted with* o-*phosphoric acid): 30 : 10 : 60% v/v. The flow rate was kept constant throughout analysis at 1 mL/min and eluent was detected at 241 nm.

Forced degradation experiments were carried out on chlorthalidone under various conditions explained in ICH guideline Q1A(R2),* namely*, acid, alkali, wet heat, dry heat, and oxidative and photolytic conditions [9]. Full factorial experimental design was used for acid and alkali forced degradation:

(a) *Acid degradation*: 1 mg/mL mixture of chlorthalidone in *X*_1_ M HCl was heated under reflux at *X*_2_°C for *X*_3_ min. Three factors, strength of acid (*X*_1_), temperature of heating (*X*_2_), and time of exposure (*X*_3_), were studied at two levels. The high level (+1) for *X*_1_, *X*_2_, and *X*_3_ was 0.1 M, 80°C, and 60 min, respectively, and the low level (−1) for *X*_1_, *X*_2_, and *X*_3_ was 0.01 M, 55°C, and 30 min, respectively. As three factors were studied at two levels, a 2^3^ factorial design was used and experimental matrix of eight experiments was prepared with combination of each factor at each level.
(b) *Alkali degradation*: 1 mg/mL mixture of chlorthalidone in *X*_1_ M NaOH was heated under reflux at *X*_2_°C for *X*_3_ min. Three factors, strength of alkali (*X*_1_), temperature of heating (*X*_2_), and time of exposure (*X*_3_), were studied at two levels. The high level (+1) for *X*_1_, *X*_2_, and *X*_3_ was 0.1 M, 80°C, and 30 min, respectively, and the low level (−1) for *X*_1_, *X*_2_, and *X*_3_ was 0.01 M, 55°C, and 15 min, respectively. As three factors were studied at two levels, a 2^3^ factorial design was used and experimental matrix of eight experiments was prepared with combination of each factor.
(c) *Wet heat degradation*: 1 mg/mL mixture of chlorthalidone in water was heated under reflux at 80°C for 48 hr.
(d) *Oxidative degradation*: 1 mg/mL mixture of chlorthalidone in 30% of H_2_O_2_ was kept under dark at room temperature for 48 hr.
(e) *Dry heat degradation*: 10 mg of chlorthalidone was spread as a thin film in Petri plate and placed in a hot air oven set at a temperature of 80°C for 24 hr.
(f) *Photolytic degradation*: chlorthalidone powder was spread as a thin layer in Petri plate and exposed to direct sunlight for 7 days.

### 2.4. Chromatographic Analysis of Forced Degraded Samples

After degradation, each sample obtained under each forced degradation condition was diluted appropriately with mobile phase to get a final concentration of 10 *μ*g/mL; the resulting solution was injected in the column under described chromatographic condition. The chromatogram obtained was studied for area of drug peak and appearance of secondary peaks. The decrease in the area of the drug peak and the occurrence of secondary peaks was considered as indication of degradation. The % degradation was calculated as

(1) %  degradation=area  of  unstressed−area  of  stressedarea  of  unstressed×100.

### 2.5. Calibration Curve

Standard stock solution of chlorthalidone was prepared in methanol to obtain a concentration of 1 mg/mL. The resulting solutions were diluted with mobile phase to get concentrations in the range of 2–12 *μ*g/mL and each solution was subjected to chromatographic analysis in triplicate under mentioned chromatographic conditions. The peak areas were plotted on *y*-axis and their respective concentrations were plotted on *x*-axis. Furthermore, the linear regression was performed to generate least square line and regression equation of type:

(2) $$\begin{array}{c} y=ax+b. \end{array}$$

### 2.6. Method Validation

Analytical method validation was carried out as per ICH method validation guideline Q2(R1) [10]. The method was validated for specificity, accuracy, precision, limit of detection (DL), and limit of quantitation (QL). Accuracy and precision were evaluated by fortifying a placebo with amounts of drug corresponding to 80%, 100%, and 120% of label claimed and analyzing the resulting mixture in triplicate over three days.

### 2.7. Analysis of Formulation

Twenty tablets were weighed and finely powdered. A quantity of powder equivalent to 10 mg of chlorthalidone was transferred to 100 mL volumetric flask and was sonicated with 80 mL methanol for 10 min. The excipients were separated by filtration and the volume was made up to the mark with the same solvent. From the resulting solution, 1 mL aliquot was removed and transferred to 10 mL volumetric flask. The volume was made up to the mark with mobile phase to get a 10 *μ*g/mL solution. The resulting solution was subjected to chromatographic analysis in triplicate. The drug peak area was referred to linear regression equation to get the sample concentration and nominal % of label claim.

## 3. Results and Discussion

### 3.1. Optimization of Mobile Phase

To get adequate retention and resolution of chlorthalidone and its formed degradation products under various forced degradation conditions, mobile phases of different strengths and pH were tried. All buffer solutions used in mobile phases were prepared as per the procedure described in Synder et al. [11]. From the chromatograms obtained, it was concluded that the mobile phase consisting of methanol : acetonitrile : 20 mM phosphate buffer (pH 3.0) (30: 10: 60% v/v) gave adequate retention of chlorthalidone and also resolved chlorthalidone from its degradation products formed under various forced degradation conditions. The maximum absorption wavelength of chlorthalidone and its degradation products was found to be 241 nm.

### 3.2. Forced Degradation Behavior of Chlorthalidone

**Figure 2:**
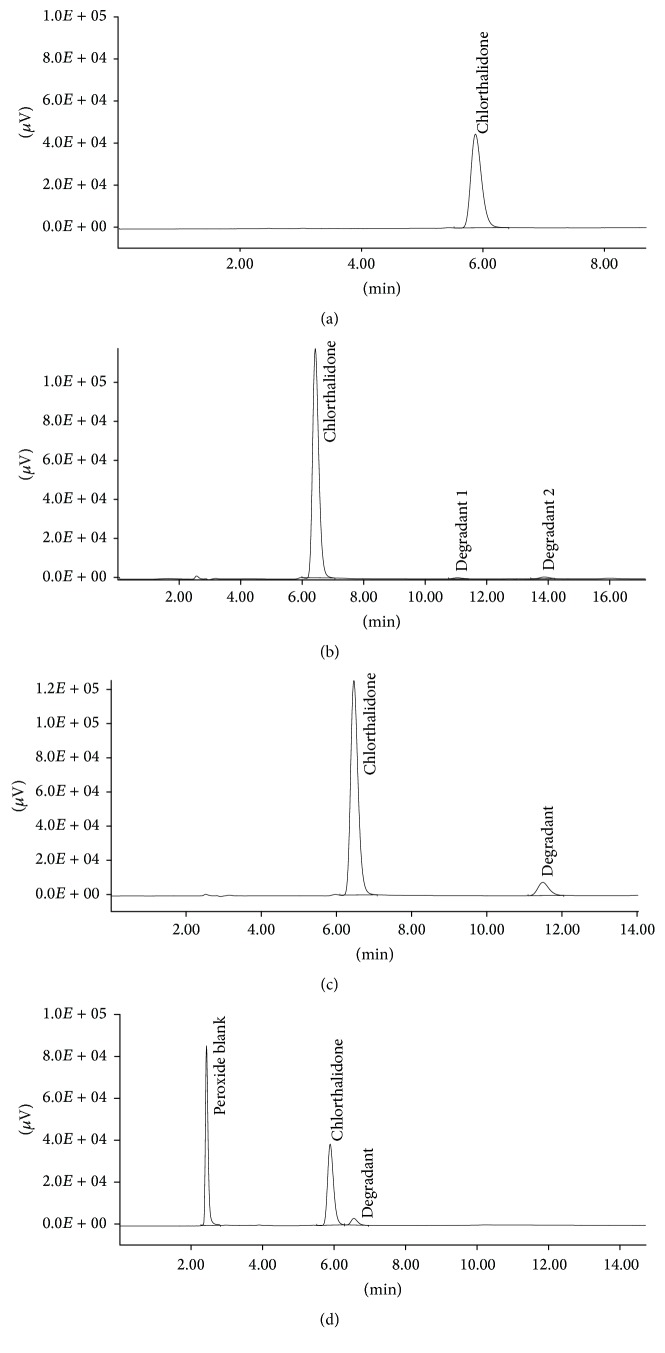
(a) Representative chromatogram of unstressed chlorthalidone. (b) Representative chromatogram of acid treated chlorthalidone. (c) Representative chromatogram of alkali treated chlorthalidone. (d) Representative chromatogram of oxidative degradation of chlorthalidone.

When forced degradation experiments were performed and subjected to chromatographic analysis, it was found that chlorthalidone was stable to wet heat, dry heat, and photolytic conditions but substantial degradation was obtained under acid, alkali, and oxidative conditions. Under acid degradation condition, two degradation products were found at 11.07 min and 13.9 min and under alkali degradation condition, one degradation product was obtained at 11.49 min. When drug was subjected to oxidative degradation condition for 48 hrs, one degradation product was obtained at 6.55 min. The chromatograms of unstressed chlorthalidone and chlorthalidone exposed to acid degradation, alkali degradation, and oxidative condition are shown in Figures 2(a), 2(b), 2(c), and 2(d), respectively.

### 3.3. Experimental Design in Acid and Alkali Forced Degradation Conditions

From the experimental matrix constructed (Table 1) as per the experimental conditions discussed for acid and alkali degradation conditions and from the % degradation obtained for each experiment, polynomial equations were obtained for acid and alkali degradation, respectively.

The general polynomial equation for 2^3^ factorial design is

(3) Y=β0+β1X1+β2X2+β3X3+β12X1X2+β23X2X3+β13X1X3+β123X1X2X3,

where *Y* is the response (% degradation), *β*_0_ is intercept, and *β*_1_, *β*_2_, and *β*_3_ are coefficients for variables *X*_1_, *X*_2_, and *X*_3_, respectively. Similarly *β*_12_, *β*_23_, *β*_13_, and *β*_123_ are the coefficients for interaction of variables, *X*_1_ and *X*_2_, *X*_2_ and *X*_3_, *X*_1_ and *X*_3_, and *X*_1_, *X*_2_, and *X*_3_, respectively.

To obtain the polynomial equations for acid and alkali degradation condition, the value of each coefficient and intercept for each degradation condition was determined.

The intercept (*β*_0_) for polynomial equation was calculated as

(4) $$\begin{array}{c} \beta_{0}=\frac{\sum_{}^{}XY}{2^{n}} \end{array}$$

and the respective coefficients were calculated as

(5) $$\begin{array}{c} \beta_{1}=\frac{\sum_{}^{}X_{1}Y}{2^{n}}, \end{array}$$

where *X*_1_ is the value of the column and *Y* is the response (% degradation of the drug).

All other coefficients were obtained in a similar manner and the polynomial equations were obtained. The polynomial equations for acid and alkali degradation were obtained as follows.

The polynomial equation for acid degradation is

(6) Y=18.32−0.67X1+10.26X2+4.56X3−0.16X1X2+2.59X2X3+0.44X1X3−0.10X1X2X3.

The polynomial equation for alkali  degradation is

(7) Y=19.64+0.87X1+10.88X2+3.08X3+0.67X1X2+0.87X2X3−0.25X1X3+0.67X1X2X3.

Furthermore, to obtain the regression of the polynomial equations (see (6) and (7)) and to determine the significant factors, Yates analysis was performed as presented in Tables 2(a) and 2(b) [12].

The regression of each polynomial equation obtained for acid degradation (see (5)) and alkali degradation (see (6)) was calculated using the following formula:

(8) $$\begin{array}{c} R^{2}=\frac{\text{Total of significant mean square}}{\text{Total of mean square}}. \end{array}$$

The regressions for acid degradation and alkali degradation polynomial equations were obtained as 0.9462 and 0.9770, respectively, and were found to be in acceptable range.

For acid degradation, when all other mean square values were divided by the average least mean square, experimental *F* values were found. When these were compared with tabulated *F* values, it was found that 3360 and 648 were significantly higher than tabulated *F* values (98.49 at *p* < 0.01). Hence it had been concluded that factor *X*_2_ (temperature) and factor *X*_3_ (time of heating) were significant.

For alkali degradation, when all other mean square values were divided by the average least mean square, experimental *F* values were found. When these were compared with tabulated *F* values, it was found that 284 was significantly higher than tabulated *F* values (98.49 at *p* < 0.01). Hence, it was concluded that factor *X*_2_ (temperature of heating) was significant.

To confirm the significant factors obtained for acid and alkali degradation experiments in Yates analysis and ANOVA, Pareto chart for Experiment versus Normalized squares was plotted. For acid degradation and alkali degradation, Normalized square values were plotted as depicted in Tables 3(a) and 3(b), respectively. The obtained values of effect for each experiment were ranked in decreasing order and Normalized square for each experiment was calculated using the following equation:

(9) $$\begin{array}{c} \text{Normalized square}=100\times \frac{E^{2}}{\sum E^{2}}. \end{array}$$

**Figure 3:**
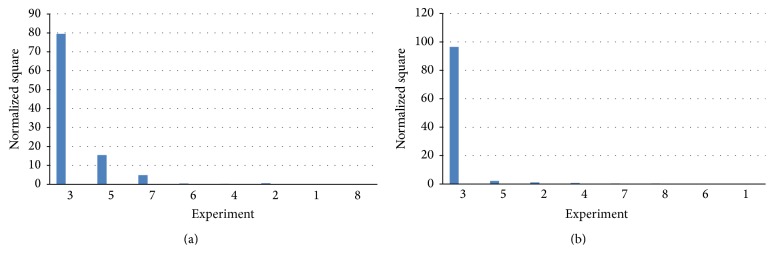
(a) Pareto chart for acid degradation. (b) Pareto chart for alkali degradation.

Furthermore, the representative Pareto charts were plotted as presented in Figures 3(a) and 3(b), for acid and alkali degradation, respectively. From the presented Pareto charts, it was concluded that the time of heating was the most significant factor followed by temperature in acid degradation and time of heating was the most significant factor in alkali degradation conditions.

To obtain optimized experimental conditions for acid degradation, surface response curve was generated with the help of polynomial equation obtained for acid degradation.

For acid degradation condition, by substituting value of *X*_1_ = 0 (average of +1 to −1) in (6) and rearranging corresponding equation for *X*_3_, the equation is obtained as

(10) $$\begin{array}{c} X_{3}=\frac{\left(Y-18.32-10.26X_{2}\right)}{4.56+2.59X_{2}}. \end{array}$$

**Figure 4:**
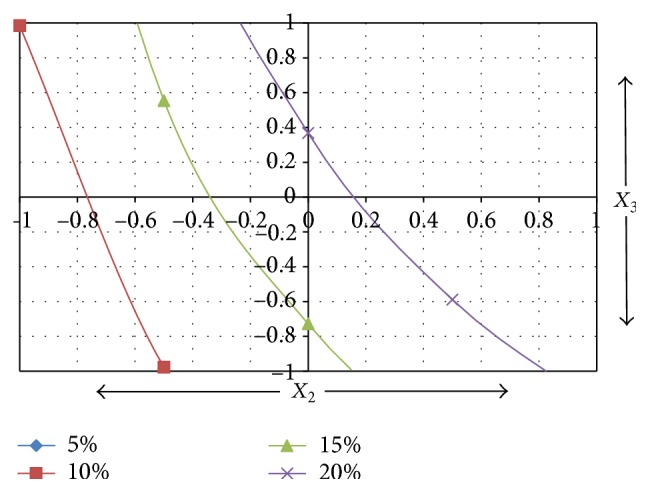
Surface response curve of transformed values for acid degradation from −1 to +1 range.

Furthermore, *Y* values (% degradation) were assumed to be 5%, 10%, 15%, and 20%; the values for *X*_3_ at various levels of *X*_2_ (−1 to +1) were calculated (Table 4) and the respective surface response curve was plotted as depicted in Figure 4.

From the surface response curve for acid degradation, it was observed that, with transformed value for *X*_2_ = −1 and *X*_3_ = +1, 10% degradation can be expected.

Thus, optimum 10% acid degradation would result when chlorthalidone was heated using 0.55 M HCl at 55°C for 60 min.

Also, for alkali degradation the actual values are obtained from transformed values using the following equation:

(11) Transformed Value=X−the average of two levelsone−half the difference of the levels.

The transformed values for *X*_2_ were obtained for 5%, 10%, 15%, and 20% of degradation, at *X*_1_ = 0 and *X*_3_ = 0.

*Y* values for 5% and 20% were obtained beyond the range of −1 to +1 experimental domain, −1.32 and 0.058, respectively. For 10% and 15% degradation, the transformed values of −0.86 and −0.44 were obtained, respectively.

The above obtained transformed values were decoded using (11). Thus, optimum 10% alkali degradation would result when chlorthalidone was heated using 0.055 M at 56.75°C for 22.5 min.

Actual experiments were performed in triplicate and subjected to chromatographic analysis. The average % degradation of three experiments was compared with the predicted response. No significant difference was observed between predicted value and observed value.

### 3.4. Calibration Curve

When calibration standards in the range of 2–12 *μ*g/mL were analyzed in triplicate and plot of peak area* versus* concentration was subjected to least square regression, the respective linear equation was

(12) $$\begin{array}{c} y=117762x+1167.7, \end{array}$$

where *x* is the concentration (*μ*g/mL) and *y* is the peak area (*μ*V). The correlation coefficient was 0.999. Student's *t*-test was performed to verify the significance of experimental intercept and slope in the regression equation. According to the results, they were not significantly different from zero and one value, respectively, for *p* > 0.05. The analysis of variance was applied to verify linearity of the method. From the result it has been observed that the calculated *F* (41454.97) was greater than the tabulated *F* (7.7) at 5% level of significance, concluding that a linear relationship exists between the peak area and concentration.

### 3.5. Method Validation

The results obtained for accuracy and precision studies are shown in Table 5. The % recovery close to 100% and the low values of % RSD suggest an acceptable accuracy of the method. Furthermore, the intraday and interday results at each level were subjected to one-way analysis of variance and *F* values for each level were determined as the ratio of between mean square (BMS) to within mean square (WMS):

(13) $$\begin{array}{c} \left(\;F=\frac{\text{BMS}}{\text{WMS}}\;\right). \end{array}$$

see [13].

The obtained *F* values were found to be less than the tabulated *F*_(2,6)_ at *α* = 0.05 (tabulated *F* value = 5.14). These indicated that there was no significant difference between intraday variability and interday variability, suggesting good intermediate precision of the method. A plot of quantity added to the quantity obtained resulted in a straight line with the slope of 1.1667 and the intercept of 0.998, encompassing 1 and 0, respectively. This indicated the linearity of the method in the selected range of 80–120% of the label claimed. Based on the SD of the response and the slope, the limit of detection (DL) was found to be 0.678 *μ*g/mL and limit of quantitation (QL) was 1.872 *μ*g/mL. The chromatograms of blank and placebo solutions showed no interfering peak at the retention time of the drug indicating specificity of the developed method.

### 3.6. Analysis of Formulation

The drug content was found to be 101.28 ± 1.17% with a % RSD of 1.16. The % RSD value indicated the suitability of the method for routine analysis of chlorthalidone in formulation.

## 4. Conclusion

The developed HPLC technique is precise, specific, accurate, and stability-indicating. Validation of the method proved that the method is suitable for the analysis of chlorthalidone in tablet formulation without any interference from common excipients or potential degradation product of chlorthalidone and excipients. The developed method can be used for routine analysis of chlorthalidone tablets or for assay of chlorthalidone tablets from stability batches.

## Figures and Tables

**Table 1:** Experimental matrix of 2^3^ factorial design for acid and alkali degradation.

Expt. number	Levels of factor in experiment			Interaction				Total	% acid deg.	% alkali deg.
	*X*_1_	*X*_2_	*X*_3_	*X*_1_ *X*_2_	*X*_2_ *X*_3_	*X*_1_ *X*_3_	*X*_1_ *X*_2_ *X*_3_			
1	−1	−1	−1	+1	+1	+1	−1	+1	7.14	5.43
2	+1	−1	−1	−1	+1	−1	+1	+1	5.03	7.66
3	−1	+1	−1	−1	−1	+1	+1	+1	22.61	25.43
4	+1	+1	−1	+1	−1	−1	−1	+1	20.27	27.69
5	−1	−1	+1	+1	−1	−1	+1	+1	10.00	11.69
6	+1	−1	+1	−1	−1	+1	−1	+1	10.07	10.25
7	−1	+1	+1	−1	+1	−1	−1	+1	36.23	32.51
8	+1	+1	+1	+1	+1	+1	+1	+1	35.24	36.42

+1 indicates the high level for each factor and −1 indicates the low level for each factor.

**(a):** 

Expt.	*Y* (response)	*A*	*B*	*C*	Effect	Mean square	*F* value
(1) (−, −, −)	7	12	55	146	—	—	—
(2) (+, −, −)	5	43	91	−6	−1.5	4.5	18
(3) (−, +, −)	23	20	−5	82	20.5	840.5	3360
(4) (+, +, −)	20	71	−1	−2	−0.5	0.5	2
(5) (−, −, +)	10	−2	31	36	9	162	648
(6) (+, −, +)	10	−3	51	4	1	2	8
(7) (−, +, +)	36	0	−1	20	5	50	200
(8) (+, +, +)	35	−1	−1	0	0	0	0

**(b):** 

Expt.	*Y* (response)	*A*	*B*	*C*	Effect	Mean square	*F* value
(1) (−, −, −)	5	13	66	156	—	—	—
(2) (+, −, −)	8	53	90	8	2	8	2.46
(3) (−, +, −)	25	22	6	86	21.5	924.5	284.46
(4) (+, +, −)	28	68	2	6	1.5	4.5	1.38
(5) (−, −, +)	12	3	40	24	6	72	22.2
(6) (+, −, +)	10	3	46	−4	−1	2	0.62
(7) (−, +, +)	32	−2	0	6	1.5	4.5	1.38
(8) (+, +, +)	36	4	6	6	1.5	4.5	1.38

**(a):** 

Rank	Expt. number	Value (*E*)	*E* ^2^	Normalized square
1	(3) (−, +, −)	20.5	420.25	79.32
2	(5) (−, −, +)	9	81	15.29
3	(7) (−, +, +)	5	25	4.71
4	(6) (+, −, +)	1	1	0.19
5	(8) (+, +, +)	0	0	0
6	(4) (+, −, +)	−0.5	0.25	0.57
7	(2) (+, −, −)	−1.5	2.25	0.42
8	(8) (−, −, −)	—	—	—

		Total	529.75	

**(b):** 

Rank	Expt. number	Value (*E*)	*E* ^2^	Normalized square
1	(3) (−, +, −)	21.5	462.25	96.52
2	(5) (−, −, +)	3	9	1.87
3	(2) (+, −, −)	2	4	0.84
4	(4) (+, +, −)	1.5	2.25	0.47
5	(7) (−, +, +)	0.75	0.5625	0.12
6	(8) (+, +, +)	0.75	0.5625	0.12
7	(6) (+, −, +)	−0.5	0.25	0.05
8	(1) (−, −, −)	—	—	—

		Total	4748.88	

**Table 4:** Transformed values (*X*_3_) at various levels of *X*_2_ for acid degradation conditions.

*X*_3_ (for *X*_1_ = 0)	5%	10%	15%	20%
−1	−1.55	0.98	3.52	6.06
−0.5	−2.51	−0.98	0.55	2.09
0	−2.92	−1.82	−0.73	0.37
0.5	−3.15	−2.30	−1.44	−0.59
1	−3.30	−2.60	−1.90	−1.2

**Table 5:** Accuracy and precision studies.

Amount added (mg)	Amount found (mg)			Within mean square	Between mean square	*F* value
	Day 1	Day 2	Day 3			
80% (10 mg)	10	10.02	10	0.0009	0.00028	0.3086
	10.05	10.03	10.09			
	10.03	10.03	10.04			
Mean	10.03	10.03	10.04			
SD	0.252	0.058	0.451			
% RSD	0.251	0.058	0.449			

100% (12.5 mg)	12.45	12.42	12.45	0.00208	0.00174	0.8396
	12.42	12.41	12.42			
	12.33	12.49	12.46			
Mean	12.4	12.44	12.44			
SD	0.62	0.44	0.208			
% RSD	0.503	0.350	0.167			

120% (15 mg)	14.09	14.01	14.032	0.00078	0.0012	1.508
	14.02	14.03	14.07			
	14.06	14.04	14.09			
Mean	14.06	14.03	14.06			
SD	0.351	0.152	0.295			
% RSD	0.251	0.110	0.209			
